# Identification of Rice Large Grain Gene *GW2* by Whole-Genome Sequencing of a Large Grain-Isogenic Line Integrated with Japonica Native Gene and Its Linkage Relationship with the Co-integrated Semidwarf Gene *d60* on Chromosome 2

**DOI:** 10.3390/ijms20215442

**Published:** 2019-10-31

**Authors:** Motonori Tomita, Shiho Yazawa, Yoshimasa Uenishi

**Affiliations:** 1 Research Institute of Green Science and Technology, Shizuoka University, 836 Ohya, Suruga-ku, Shizuoka City, Shizuoka 422-8529, Japan; u.yoshimasa0202@gmail.com; 2 Faculty of Agriculture, Tottori University, 4-101 Koyama Minami, Tottori 680-8550, Japan; 8as41.yazawa@gmail.com

**Keywords:** rice, large grain gene, large grain-isogenic Koshihikari, fine mapping, NGS, *GW2*, co-integration, gene recombination, semidwarf gene, *d60*, linkage, chromosome 2

## Abstract

Genetic analysis of “InochinoIchi,” an exceptionally large grain rice variety, was conducted through five continuous backcrosses with Koshihikari as a recurrent parent using the large grain F_3_ plant in Koshihikari × Inochinoichi as a nonrecurrent parent. Thorough the F_2_ and all BCnF_2_ generations, large, medium, and small grain segregated in a 1:2:1 ratio, indicating that the large grain is controlled by a single allele. Mapping by using simple sequence repeat (SSR) and single nucleotide polymorphism (SNP) markers with small grain homozygous segregants in the F_2_ of Nipponbare × Inochinoichi, revealed linkage with around 7.7 Mb markers from the distal end of the short arm of chromosome 2. Whole-genome sequencing on a large grain isogenic Koshihikari (BC_4_F_2_) using next-generation sequencing (NGS) identified a single nucleotide deletion in *GW2* gene, which is located 8.1 Mb from the end of chromosome 2, encoding a RING protein with E3 ubiquitin ligase activity. The *GW2*-integrated isogenic Koshihikari showed a 34% increase in thousand kernel weight compared to Koshihikari, while retaining a taste score of 80. We further developed a large grain/semi-dwarf isogenic Koshihikari integrated with *GW2* and the semidwarfing gene *d60*, which was found to be localized on chromosome 2. The combined genotype secured high yielding while providing robustness to withstand climate change, which can contribute to the New Green Revolution.

## 1. Introduction

There is a demand for a dramatic increase in the production of rice, as it is a staple food for over half of the world’s rapidly increasing population. In the period of the 20th century when most rice breeding took place, known as the “Green Revolution,” improving rice stems to be shorter decreased the likelihood of plant lodging, which made heavy manuring and dense planting possible, and radically increased yields. The “semidwarfness” led to a twofold increase in rice yields worldwide between the 1960s and the 1990s [1]. However, the trend of rice productively has now begun to plateau [2]. On top of that, the suppression of stem length is dependent on a single semidwarfing gene called *sd1*. In preparation for future increases in population and risk of crop damage due to climate change, there is a renewed demand for a “New Green Revolution” for genetic improvements to increase yields and make rice plants more robust.

Grain size is a major factor in affecting rice yield. Six quantitative trait loci (QTLs) genes related to rice grain size have been isolated [3,4,5,6,7,8]. For four of these genes, a loss of function causes the grains to be bigger, which shows that there is a mechanism that suppresses grain size. In general, Japonica rice has short and round grains, while Indica rice has long and thin grains. Whole-genome analysis of cultivated rice and wild rice shows that the domestication of rice started near the middle of the Pearl River in China, when Japonica was derived from a population of *Oryza rufipogon* [9]. Subsequently, Indica arose from hybrids between strains of wild rice in Southeast Asia and South Asia and Japonica. The *qSW5* and *GS3* genes confer grain size traits in Japonica and Indica, respectively [5,9].

The japonica rice Koshihikari is the leading variety in Japan, accounting for 36.1% of rice acreage in the country. The patent on the plant variety protection of Koshihikari, which was registered in 1956, has expired; therefore, global competition with Koshihikari produced in foreign countries is now of concern. The 2016 Trans-Pacific Partnership (TPP) eliminated tariffs on 82% (2135) of the 2594 agriculture, forestry, and fishery products that are imported by Japan [10]. The 341 JPY/kg (140%) tariffs on rice were maintained, but the simultaneous buy-and-sell (SBS) tender system with the US and Australia, which already produce Koshihikari, provides a special import framework for 78,400 t of rice. Foreign-produced Koshihikari is roughly 35% less expensive than Japanese Koshihikari, and it is genetically the same, with no difference in taste. Consequently, the influx of inexpensive Koshihikari produced in foreign countries is a concern to Japan. Furthermore, after the Trump administration began, the US pulled out of the TPP and has been pushing for a Trade Agreement on Goods (TAG) between the US and Japan to take its place. In the future, rice trade will inevitably be liberalized. Moreover, if Japan’s self-sufficiency collapses, the country will lose its paddy fields, which would not be ideal for maintaining national land conservation. In order to resolve this critical situation, there is a need to develop a low-cost and high-yield “super Koshihikari” variety that can compete in the international market.

Intensified climate change due to global warming is causing damage to crops on a global scale. Global contributions due to the New Green Revolution could uphold the innovation policy. In 2018, Japan was hit with the Western Japan heavy rain and floods [11], and seven large typhoons with wind speeds over 54 m/s, which were the worst in Japan’s history [12]. Typhoons Jebi and Trami were equivalent to the Isewan Typhoon. These extreme weather phenomena have caused marked damage to agriculture, forestry, and fisheries (totaling 436.5 billion yen) [13]. There is a need to genetically improve the sturdiness and robustness of rice plants to withstand the intensified climate change [14,15] and improve rice for the global market by lowering costs and increasing yield.

The grain weight of a large grain variety, “Inochinoichi,” is approximately 1.5 times that of Koshihikari. However, the genetic mode of the large grain is unclear, so it is not used for plant breeding at all. The production of “Inochinoichi” is also limited to the area around Gifu Prefecture. If the causative gene for the large grain of inochinoichi were to be identified, its possibility of application to improve varieties, including Koshihiakri, or to develop new varieties would be expanded. In this study, we identified from this unused and buried genetic resource Inochinoichi, the gene responsible for the large grain, and then by applying the gene to develop a large-grain Koshihikari. First, we conducted genetic analysis of the large grain through five continuous backcrosses with Koshihikari as a recurrent parent using the large grain segregant in the F_2_ generation of a Koshihikari × Inochinoichi as a nonrecurrent parent. Then, we conducted whole-genome analysis of the developed large grain isogenic Koshihikari and identified a gene responsible for the large grain. Furthermore, we developed a large grain/semi-dwarf isogenic line by integrating both the identified large grain gene and the semidwarfing gene *d60* [16].

## 2. Results

### 2.1. Inheritance and Phenotypic Expression of Large Grain Gene in an Isogenic Background

**Figure 1:**
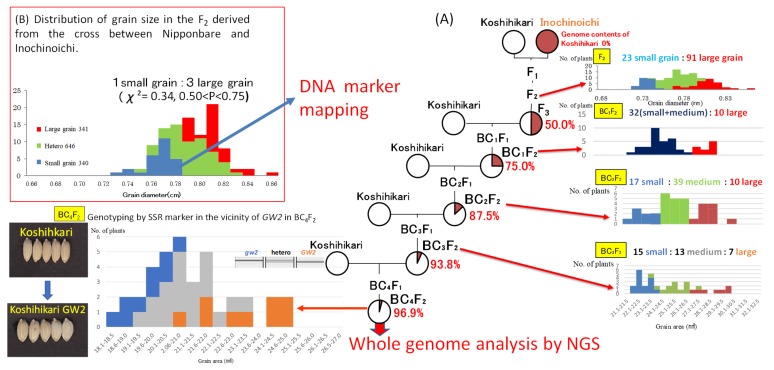
Procedure to identify large grain gene in Inochinoichi. (**A**) Whole-genome analysis of isogeneic line developed by continuous back cross integrating the large grain gene. Genetic analyses for large grain through the four times of backcrosses with Koshihikari, each BC_2_F_2_ to BC_4_F_2_ progeny segregated in the theoretical ratio for single incomplete dominance gene, namely 1 large grain:2 medium grain:1 small grain. This indicates that the large grain is definitely inherited as a single allele. (**B**) Molecular linkage analysis by using small grain homozygous F_2_ derived from the cross of Nipponbare × Inochinoichi.

**Figure 2:**
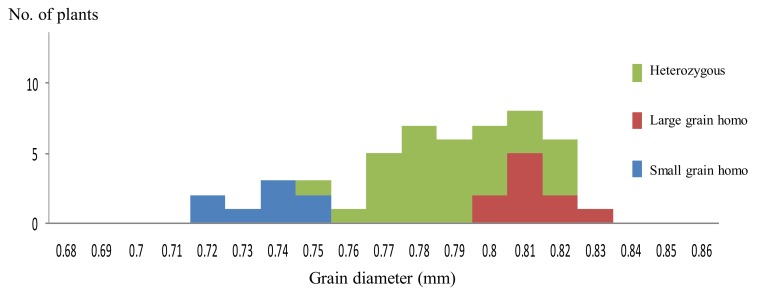
Distribution of mean value of grain diameter in 50 F_3_ line derived from the cross, Koshihikari × Inochinoichi. F_3_ lines segregated in a ratio of 10 large grain homozygous lines:32 heterozygous lines:8 small grain homozygous lines, which fit to the theoretical single gene ratio.

As shown in Figure 1A, in the F_2_ generation of Koshihikari × Inochinoichi, grain diameters showed a bimodal distribution in 0.69–0.85 mm, which were comparable to both parenteral range. Namely large grain plants with grain diameters of 0.78–0.85 mm, the same as Inochinoichi, and small grain plants with grain diameters of 0.69–0.77 mm the same as Koshihikari were segregated in a ratio of 134 large grains:52 small grains, which fit to a 3:1 ratio, (χ^2^ = 0.87, df = 1, 0.35 < P < 0.40). Next, we conducted a progeny test using 50 F_3_ lines consisting of 50 randomly selected F_2_ plants of Koshihikari × Inochinoichi. As a results, the mean values of grain size in each F_3_ line were distributed as shown in Figure 2. Namely, the F_3_ progeny of large grain F_2_ plants (grain diameter: 0.78–0.85 mm) were classified into a line that had fixed in large grains with diameters of 0.8–0.83 mm and a line that segregated within the lines, whereas the F_3_ progeny of small grain F_2_ plants (grain diameter: 0.68–0.77 mm) fixed in small grain with diameters of 0.72–0.75 mm. In other words, F_3_ lines segregated in a ratio of 10 large grain homozygous lines:32 heterozygous lines:8 small grain homozygous lines, consistent with the theoretical single gene ratio (χ^2^ = 4.08, df = 1, 0.10 < P < 0.25). Using the fixed large grain homozygous plant in the F_3_ generation (grain diameter: 0.75 mm) as a nonrecurrent parent, five times of continuous backcrosses with Koshihikari as a recurrent parent were conducted. The BC_1_F_2_ plants segregated in a ratio of 10 large grain plants (grain area: 24.6–25.9 mm^2^):32 small and medium grain plants (grain area: 20.6–24.0 mm^2^) (Figure 1A). Furthermore, a large grain segregant in the BC_1_F_2_ generation (grain area: 25.9 mm^2^) was used in a second backcross with Koshihikari, which yielded a BC_2_F_2_ plants segregated in a ratio of 17 large grain plants (grain area: 23.6–25.1 mm^2^):39 medium plants (grain area: 20.6–23.5 mm^2^):14 small grain plants (grain area: 19.6–20.5 mm^2^); in both generations, segregation ratios fit to the theoretical single gene ratio (χ^2^ = 0.03, df = 1, 0.50 < P < 0.90; χ^2^ = 1.17, df = 2, 0.55 < P < 0.60) (Figure 1A). Subsequently, the BC_3_F_2_ plants segregated in a ratio of 15 large grain (grain area: 19.6–20.5 mm^2^):13 medium grain (grain area: 19.6–20.5 mm^2^):7 small grain (grain area: 19.6–20.5 mm^2^) (χ^2^ = 5.97, df = 2, 0.05 < P < 0.10). Next, a large grain BC_3_F_2_ segregant (grain area: 23.05 mm^2^) was used for the fourth backcross with Koshihikari, whose BC_4_F_2_ progenies segregated in a ratio of 8 large grain (grain area: 26.1–29.5 mm^2^):18 medium grain (grain area: 23.6–26.0 mm^2^):10 small grain (grain area: 21.1–23.5 mm^2^) plants, that fit well to a 1:2:1 ratio (χ^2^ = 0.20, df = 2, 0.85 < P < 0.90) (Figure 1A). As seen above, from genetic analyses of large grain through the four times of backcrosses with Koshihikari, each BC_2_F_2_ to BC_4_F_2_ progeny segregated in the theoretical ratio for single incomplete dominance gene, namely 1 large grain:2 medium grain:1 small grain (Figure 1A). This indicates that the large grain is definitely inherited as a single allele. Finally, the large grain isogenic Koshihikari (BC_5_F_2_), which produced by backcrossed with Koshihikari and a large grain BC_4_F_2_ segregant, showed a grain area 27.7% greater than that of Koshihikari (Koshihikari average grain area: 22.32 mm^2^, large grain phenotype average: 28.50 mm^2^), and the thousand kernel weight increased by 34%. Its taste score (80.0) was also equivalent to that of Niigata Koshihikari (81.0) (Table 1); thus, this isogenic Koshihikari holds promise as a Super Koshihikari, which is distinguishable from US-made Koshihikari.

### 2.2. Candidate Region of Large Grain Gene

**Figure 3:**
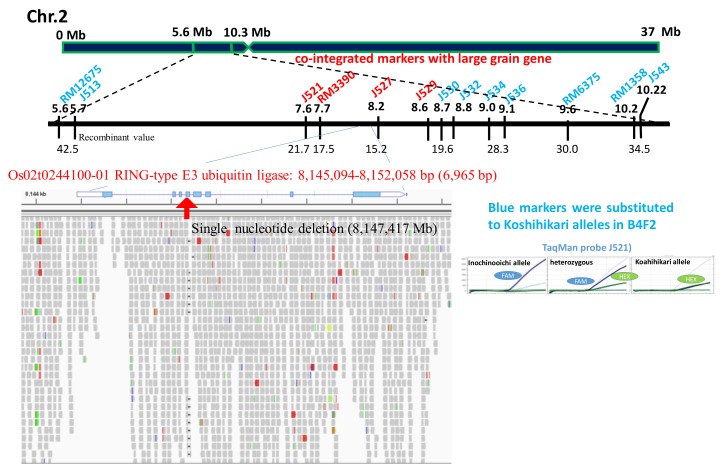
Identification of single nucleotide deletion in *GW2* responsible for large grain size of Inochinoichi. SNP allele-specific TaqMan probes were designed and labeled using the fluorescent dyes FAM or HEX. The real-time polymerase chain reaction (PCR) was used to amplify the allele-specific fluorescence. Blue DNA markers were substituted to Koshihikari alleles in B_4_F_2_. However, red DNA markers were inherited together with *GW2*.

Using small grain homozygous F_2_ segregants of a Nipponbare × Inochinoichi (Figure 1B), we genetically mapped the large grain gene by SSR and SNP markers across rice’s 12 chromosomes. Our results showed that the recombinant values between DNA markers and the large grain gene were detected on chromosome 2. Namely, from the distal end of the short arm of chromosome 2, 21.7 at J521 (7.6 Mb), 17.5 at RM3390 (7.7 Mb), 15.2 at J527 (8.2 Mb), 19.6 at J529 (8.6 Mb), 28.3 at J536(9.1 Mb), 30.0 at RM6375 (9.6 Mb), and 34.5 at RM1358 (10.2 Mb), respectively (Figure 3). The RM3390-homozygous plant by the diagnosis, which is linked with *GW2*, showed that the mean grain area with Inochinoichi alleles was 23.3 mm^2^, which is larger than 20.0 mm^2^ in Koshihikari (Figure 1A).

### 2.3. Identification of DNA Variation Responsible for Large Grain Using Next-Generation Sequencing (NGS)

The read sequences of Koshihikari obtained by NGS were mapped using the Nipponbare genome as a reference sequence. The cover ratio was determined to be 99.05% and the mean depth was 32.43; Finally, a 372,912,445 bp long consensus sequence of the Koshihikari genome was constructed. Next, read sequences gained from the large grain isogenic Koshihikari (BC_4_F_2_) were mapped using the consensus sequence of Koshihikari as a reference sequence. In total, 187,159,213 reads were mapped, with a mapped read rate of 99.90%, a mean read length of 123.3 bp, and a 30.95× genome coverage.

Whole-genome sequencing detected a single nucleotide deletion (adenine) from the Koshihikari genome at the 8,147,417 bp position from the distal end of the short arm of chromosome 2 (Figure 3). This was the same as a single nucleotide deletion reported in the fourth exon of *GW2* (Os02g024410), the QTL gene responsible for grain width in the large grain Chinese rice WY3 [4]. *GW2* encodes a RING protein with E3 ubiquitin ligase activity, and a frame shift caused by a nucleotide deletion in this gene causes a loss-of-function [4]. *GW2* derived from Inochinoichi is 6,965 bp with 100% identical to that of WY3, i.e., a single deletion in the fourth exon. There are two SNPs that flank the coding region of the hydroquinone glucosyltransferase gene (Os02g0242900). Our results show that the gene responsible for the large grain size of Inochinoichi, a promising gene source for increasing yield, is *GW2*. DNA markers around *GW2*, namely from the distal end of the short arm of chromosome 2, RM12675(5.6 Mb), J513(5.7 Mb), J530(8.7 Mb), J532(8.8 Mb), J534(9.0 Mb), J536(9.1 Mb), RM6375 (9.6 Mb), and RM1358 (10.2 Mb) were substituted to Koshihikari alleles in the B_4_F_2_ (Figure 3). On the contrary, Inochinoichi alleles of J521 (7.6 Mb), RM3390 (7.7 Mb), J527 (8.2 Mb), J529 (8.6 Mb) were tightly inherited together with *GW2*.

### 2.4. Linkage Relationship Between Semidwarfing Gene d60 and Large Grain Gene GW2

**Figure 4:**
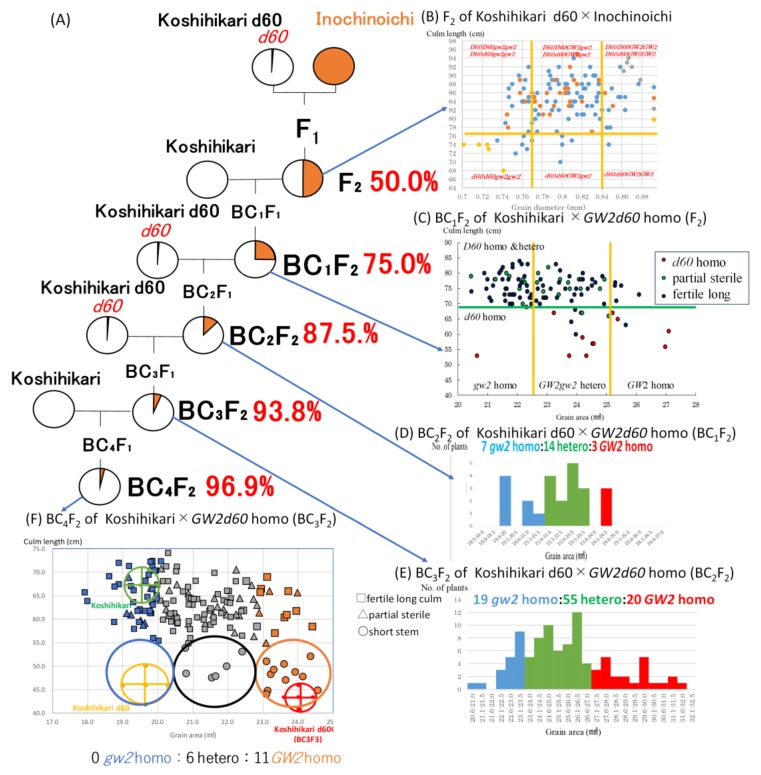
Linkage relationship between *GW2* and *d60* identified by the deviated segregation ratio through the continuous backcross process by using Koshihikari or Koshihikari *d60* as recurrent parents. (**A**) Backcross procedure combining *d60* and *GW2*. (**B**) Distribution of grain size and culm length in the F_2_ of Koshihikari d60 × Inochinoichi. (**C**) Deviated segregation for grain size in each *d60/D60* genotype in BC_1_F_2_. (**D**,**E**) Mendelian segregation for grain size in the *d60* homo background in BC_2_F_2_ and BC_3_F_2_. (**F**) Recombination value (17.6) between *GW2* and *d60* was by segregation ratio in *GW2* allele in d60 homozygous in BC_4_F_2_.

The first backcross with Koshihikari was conducted with a large grain semi-dwarf plant (stalk length: 76 cm, grain diameter: 0.8 mm) as the nonrecurrent parent segregated in the F_2_ between Koshihikari d60 (which was developed by integrating the Hokuriku 100-derived semidwarfing gene *d60* into the Koshihikari genome through seven times of backcrosses) and Inochinoichi (Figure 4A,B). Here, regarding the genetics of *d60*, in the F_1_ hybrid (genotype *D60d60Galgal*) of Koshihikari (*D60D60galgal*) × Koshihikari d60(*d60d60GalGal*), male and female gametes having both *gal* and *d60* become gamete lethal and the pollen and seed fertility decrease to 75%. As a results, the F_2_ progeny shows a unique mode of inheritance that is segregated into a ratio of 6 fertile long-culm (4*D60D60*:2*D60d60GalGal*: 2 partially sterile long-culm (*D60d60Galgal* = F_1_ type):1 dwarf(*d60d60GalGal*) [16] (Figure S1). In this study in the BC_1_F_2_ of Koshihikari/(Koshihikari d60 × Inochinoichi F_2_), the genotypic ratio for the *D60*/*d60* allele was 11 *d60* homozygous:26 partially sterile:75 long stem, which fit the theoretical ratio of 1:2:6 well (χ^2^ = 0.22, df = 2, 0.85 < P < 0.90) (Figure 4C). However, in the relationship between grain area and stem length, this contrasts with the Koshihikari*1/Koshihikari/Inochinoichi BC_1_F_2_, where there was an extremely small number of large grain segregants, a large number of small grain segregants, and no large grain long-stem segregants (Figure 4C). In other words, for BC_1_F_2_ as a whole, the ratio of (*GW2* homozygous + heterozygous): *gw2* homozygous was 73:39. This ratio should be close to 5:4, which arises when *GW2* is completely linked with *D60*. Furthermore, while the segregation of the *GW2* allele in *d60* homozygous semi-dwarf plants was 10:1 for (large grain *GW2* homozygous + hetero): small grain *gw2* homozygous, in long-stem plants, the ratio of (*GW2* large grain homozygous + heterozygous): small grain *gw2* homozygous was 63:38. In other words, while large grain plants appeared at a higher rate in the semidwarf phenotype plants, they appeared at a lower rate in the long-stem phenotype plants (Figure 4C). Considerably deviated segregation in the *GW2* locus occurred while opposing to each genotype of *d60* allele. Furthermore, if *GW2* and *d60* are inherited independently, then the appearance rates of *GW2* homozygous long stem plants and that of *GW2* homozygous long stem partially sterile plants should be 6/36 (=(4*D60D60* + 2*D60d60*)/9 × 1*GW2GW2*/4)) and 2/36(=2*D60d60*/9 × 1*GW2GW2*/4), respectively. However, actually there were no *GW2* homozygotes among long stem or long stem partially sterile plants, respectively (Figure 4C). Thus, the fact that the segregation of the *GW2* allele was considerably deviated in each genotype of *d60* suggests linkage between *GW2* and *d60*.

A large grain semi-dwarf segregant in BC_1_F_2_ was backcrossed with Koshihikari d60 as a nonrecurrent parent to make Koshihikari d60//Koshihikari/((Koshihikarid60 × Inochinoichi)F_2_) BC_2_F_2_, which segregated in a ratio of 3 large (grain area: 24.1–24.5 mm^2^):14 medium:7 small (grain area: 19.6–22.0 mm^2^) grain phenotypes = 1:2:1(χ^2^ = 2.00, df = 2, 0.30 < P < 0.50) (Figure 4D). Then, a *GW2d60* homozygous large grain semi-dwarf segregant in the BC_2_F_2_ was thirdly backcrossed with a Koshihikari d60 to make Koshihikari d60*2//Koshihikari/((Koshihikarid60 × Inochinoichi)F_2_)BC_3_F_2_, which segregated in a ratio of 20 large (grain area: 27.1–32.5 mm^2^):55 medium:19 small (grain area: 20.6–23.5 mm^2^) grain phenotypes well fit to a 1:2:1 ratio (χ^2^ = 2.74, df = 2, 0.20 < P < 0.30) (Figure 4E). Above all, the affect of the linkage between *GW2* and *d60* disappeared in the *d60* homozygous genetic background, so the large grain *GW2* locus segregated according to a Mendelian ratio of 1 *GW2* homozygous:2 heterozygous:1 *gw2* homozygous.

**Figure 5:**
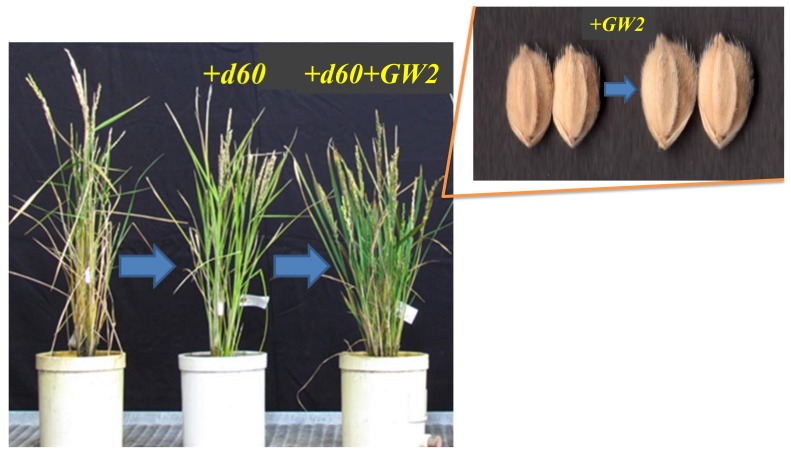
Phenotypic alteration of isogenic Koshihikari by integration of *d60* and *GW2*, derived from “Inochinoichi”.

As above, a large grain semi-dwarf plant (BC_3_F_2_), namely Koshihikari d60 integrated with *GW2*, was fourthly backcrossed with Koshihikari to make a Koshihikari///Koshihikari d60*2//Koshihikari/((Koshihikari d60 × Inochinoichi)F_2_ Gg genotype) BC_4_F_2_ generation (Figure 4A). The distribution of stem length in the BC_4_F_2_ was 17 (semi-dwarf 44.1–53.9 cm):36 (partially sterile):104 (long stem 53.9–74.1 cm) ≈ 1*d60d60GalGal*:2 *D60d60Galgal*:6(1*D60D60GglGal* + 2*D60D60Galgal* + 1*D60D60galgal* + 2*D60d60GalGal*)(χ^2^ = 0.01, df = 2, 0.9 < P < 0.95) (Figure 4F). Additionally, in each genotype for stem length, large (grain area: 22.9–24.6 mm^2^), medium (grain area: 20.1–22.8 mm^2^), and small grain (grain area: 17.9–20.0 mm^2^) were segregated in the ratio of 11 large grain:6 medium grain:0 small grain in the short stem phenotype, 4 large grain:23 medium grain:9 small grain in the partially sterile phenotype, and 10 large grain:60 medium grain:34 small grain for the long stem phenotype. In other words, as is the case in the F_2_ generation, while large grain genotypes segregated at a high rate in the *d60* homozygous semi-dwarf plants, the small grain genotypes segregated at a high rate in the long stem plants; this indicates a linkage relationship between *GW2* and *d60*. The recombinant value was calculated from the segregation ratio of 11 *GW*2 homozygous:6 *GW2gw2*:0 *gw2* homozygous plants in the semi-dwarf phenotype, as follows: ((0 × 2 small grain + 6 heterozygous specimens)/17 × 2 total semi-dwarf specimens) × 100 = 17.6 (Figure 4F). The grain weight of the *GW2d60* homozygous large-grain semidwarf isogenic line (BC_4_F_2_) increased by approximately 31% over that of Koshihikari, and the stem length decreased by 26% (Table 1, Figure 5). The robust and high yielding isogenic Koshihikari line developed in this study by integration with *d60* and *GW2* is a new rice variety could bring about the “New Green Revolution” and overcome difficulties in this age of climate change and globalization.

## 3. Discussion

Crops in Japan and the world are being damaged by climate change caused by global warming. In 2017, the Japanese government put an innovation policy into place to contribute to the world through the development of high-yield crops for a “New Green Revolution.” The rice variety “Inochinoichi” is highly rated at both the consumer and producer levels, but the genes that control its large grain size have not been elucidated, so the buried genetic resources never have been used in breeding. In order to identify the genes that control large grain characteristics in Inochinoichi, we crossed a large grain rice variety with Koshihikari and, then, conducted a backcross with Koshihikari, which showed that the large grain size is controlled by a single gene. At the same time, we conducted a linkage analysis of a large grain gene using SSR markers and SNP markers across the 12 rice chromosomes that show polymorphisms between Nipponbare and Inochinoichi. As a result, we detected a linkage between the large grain gene and a DNA marker located 7.7 Mb from the distal end of the short arm of chromosome 2. Furthermore, we established a large grain isogenic line through five continuous back crosses with Koshihikari as the recurrent parent and then analyzed its whole genome using next-generation DNA sequencing. We successfully identified the target gene for large grains integrated in the genetic background of Koshihikari. There was no public information on the consensus sequence of Koshihikari, thus, we conducted a high-coverage whole-genome analysis, by first determining a consensus sequence for Koshihikari. We found that the responsible mutation for the large grain is a single nucleotide deletion located at 8.1 Mb from the distal end of the short arm of chromosome 2, in the *GW2* gene. *GW2* was identified as the causative gene at a QTL involved in grain width in large grain Japonica rice WY3; in Indica rice FAZ1, this allele (Os02g024410) encodes a new RING protein with E3 ubiquitin ligase activity [4]. E3 ubiquitin ligase is involved in the breakdown of proteins in the ubiquitin proteasome pathway [17]. RING-type E3 ubiquitin ligase can control seed development by catalyzing the ubiquitination of expansin-like 1 (EXPLA1), a cell wall-loosening protein that increases cell growth [18]. In contrast, the *GW2* gene in WY3 lost function due to a frame shift caused by a single nucleotide deletion. It has long been known that the size of the awn covering the grain is one of the factors determining grain size [19]. In a FAZ1 near-isogenic line with *GW2* from WY3 rice, the width of the awn was extended by 26.2% because of the increased cell number [4]. In this study, *GW2,* which was identified as a loss of function by the single nucleotide deletion, increased grain weight by 34% in the genetic background of Koshihikari.

In addition, it has been shown, by isolating genes affecting grain size, that cell number of awn is a factor that determines grain size and that grains get larger due to the loss-of-function of such genes [3,4,5,6,7,8]. *GS3* (Os03g0407400), the first gene to be reported, encodes a transmembrane protein consisting of 232 amino acids [3]. It was shown through a functional complementation test that a loss-of-function by a mutation in the second exon caused an increase in grain size [20]. A gene coding for a new nuclear protein, *qSW5* (Os05g0187500), makes grain width thin in Indica rice Kasalath [5]. The *qSW5* allele in Kasalath reduces cell number in the width direction, which narrows the awn, and in turn suppresses the elongation of endosperm cells, resulting in narrowed grain width. On the contrary, the Nipponbare *qSW5* allele has a 1212 bp deletion in its coding region, which results in a loss-of-function and allows for increase in grain width. *GS5* (Os05g0158500) found in Indica rice Zhenshan97 is a regulatory factor, which encodes serine carboxypeptidase and controls positively for grain size [6]; the difference of *GS5* expression affects grain size. *GW8* derived from a high-yielding rice variety HJX74 is responsible for the QTL involved in grain width [7]; the HJX74 allele also increases grain width. *GW8* is OsSPL16 (Os08g0531600), a gene that codes for a protein that positively controls cell proliferation. The *GW8* allele in HJX74 also increases cell number, enlarging the awn and, consequently, causing increase in grain size. These past studies have shown that loss-of-function mutations such as *GW2*, *GS3*, *GS5*, *qSW5*, and *GW8* cause an increase in cell number and subsequently, cause larger grain size.

*TGW6* (Os06g0623700), a gene that increases the thousand kernel weight of Kasalath, has also been isolated [8]. *TWG6* increases the cell number in the endosperm. The *twg6* allele in Nipponbare codes for a protein that hydrolyzes indole-3-acetic acid (IAA)-glucose and synthesizes IAA, which promotes transition into the cell division stage. The Nipponbare *tgw6* allele reduces the cell number in the endosperm as well as grain length. The *TGW6* allele in Kasalath has a loss-of-function due to a single nucleotide deletion in its coding region, which means that grain length suppression via IAA does not occur, and grains elongate into a long phenotype characteristic of Indica rice. The distribution of *TGW6* was investigated in the genetic stock and the Kasalath *TGW6* allele was only found in one line of *Oryza perennis* and four local varieties in Indonesia [8]. This indicates that *TGW6* was not a target of selection and thought to have been discarded during the domestication process. The Japanese large grain rice variety Oochikara is reported to have the same *GW2* allele as those of WY3 and Inochinoichi. However, there is no historical relationship between Inochinoichi and Oochikara through their pedigree. Consequently, *GW2* is thought to be an extremely rare allele.

As discussed earlier, large grain genes that confer Indica rice its characteristics have been identified in Indica or Chinese varieties. However, breeding to enlarge grain size has never been fully explored in the Japanese leading variety Koshihikari. We now have the opportunity to utilize a large grain gene that was ignored during the domestication process to develop a high-yield rice variety. In our study, we showed evidence that an isogenic large grain Koshihikari integrated with *GW2* derived from Japanese rice via five times of backcrosses with Koshihikari has a 34% increased grain size compared to Koshihikari, and a taste score of 80.0, which is comparable to that of Niigata Koshihikari (81.0). Grain size-enlarged Koshihikari has the potential to become advantageously differentiated from US-made Koshihikari.

Rice yields around the world have doubled through the breeding of semi-dwarf varieties, which are representative of the Green Revolution in the mid-20th century, but the increase in yield is now leveling off. Additionally, there has been increased damage from lodging caused by severe weather events like the Western Japan floods and multiple typhoons under the recently intensified climate change; thus there is a need to develop rice plants that are sturdier and more robust. Also, with market liberalization through the Comprehensive and Progressive Agreement for Trans-Pacific Partnership (CPTPP) and negotiations for a Trade Agreement on Goods (TAG), there will soon be international competition in the rice market, so there is a need for low-cost and high-yielding rice. Thus, in order to make a breakthrough in high-yield breeding, which is dependent on a conventional semidwarfing gene *sd1*, we believe that to give rise to the New Green Revolution, semidwarfing should be used as a foundation with integrating/addition of genes related to high-yield including large grain and increased biomass. In this study, we combined the novel semidwarfing gene *d60* and the large grain gene *GW2* in the isogenic background of Koshihikari. In the BC_4_F_2_ by a cross Koshihikari × Koshihikari d60Gg (BC_3_F_2_), gametes with both *d60* and the gametic lethal gene *gal* are not viable, so the segregation ratio was (1*D60D60GglGal* + 2*D60D60Galgal* + 1*D60D60galgal* + 2*D60d60GalGal*):2*D60d60Galgal*:1*d60d60GalGal*. Through this genetic process, a linkage between *d60* and *GW2* on chromosome 2 was discovered with a recombination value of 17.6, according to the deviated segregation ratio of the large grain allele, namely 11 *GW2* homozygous:6 *GW2gw2*:0 *gw2* homozygous in the semidwarf *d60Gal* homozygotes. The integration of *GW2* and *d60* resulted in a 20.0% increase in grain size and a 19.2 cm reduction in stem length compared to Koshihikari, which is effective to reduce the lodging risk that accompanies the increased panicle/grain weight. We obtained genetic achievement of the effective integration of genes for large grain and robustness. We made a breakthrough in breeding, which conventionally relied only on a single gene *sd1*, by combining the semidwarfing gene with a gene for a high yields-related factor. Such a combined genotype could secure high yields while providing robustness required to withstand climate change. In other words, this idea could contribute to the New Green Revolution. We have designated the large grain isogenic line with a 34% increased grain weight due to *GW2*, and the large grain semi-dwarf isogenic line due to *GW2* + *d60*, which is capable of stable production with 31% increased grain weight/20 cm (26%) reduction in stem length, as “Koshihikari Suruga Gg” and “Koshihikari Suruga d60Gg”, respectively [21,22]. The two lines have been applied for plant variety registration. The taste and grain quality of these new varieties compared favorably with Niigata Koshihikari.

In China, grain size is being increased through the knockout of *GW2* by genome editing [23]. On the contrary, in the countries under the ratification of the Cartagena Act, including European countries and Japan, there are barriers to the social implementation of genetically modified plants. In our study, we developed a large grain semi-dwarf isogenic variety for stable production that withstands climate change though smart breeding. This was done by identifying the gene responsible for large grain size by NGS and, then, integrating it into the reference Koshihikari genome by continuous backcrossing, to finally construct a targeted gene-integrated isogenic genotype. The variety “Koshihikari Suruga d60Gg” has an epoch-making phenotype as it integrates the large grain gene *GW2*, which increases grain weight by 34%, as well as the semidwarfing gene *d60*, which reduces lodging risk, into the Koshihikari genome. This new variety could potential be a Super Koshihikari that could replace the leading variety Koshihikari which currently has a 36% share but suffers from considerable damage by abnormal weather. Our breakthrough rice plant type that integrates both semidwarfing and large grain phenotype should be a key resource for the New Green Revolution.

## 4. Materials and Methods

### 4.1. Genetic Analysis

We focused on the large grain characteristics of Inochinoichi as a genetic resource for high yields. First, we analyzed the mode of inheritance of grain size in the F_2_ generation of Koshihikari × Inochinoichi. Furthermore, we conducted a progeny test using 50 F_3_ lines derived from 50 randomly selected F_2_ plants from Koshihikari × Inochinoichi. We then conducted five times of backcrosses with Koshihikari as a recurrent parent by using a large grain homozygous F3 plant (grain diameter: 0.75 mm) of Koshihikari × Inochinoichi as a nonrecurrent parent. We conducted genetic analysis of large grain in each BCnF_2_ generation through five times of backcrosses, and the large grain homozygous segregants in each BCnF_2_ were used as pollen parents for backcrosses with Koshihikari.

In order to develop an isogenic line that is both a semi-dwarf and large grains, the large grain semidwarf segregant in the F2 generation of Koshihikari d60 × Inochinoichi was used as a nonrecurrent parent to backcross with Koshihikari once, then Koshihikari d60 twice, then Koshihikari once again. Koshihikari d60 is an isogenic Koshihikari integrated with semidwarfing gene *d60* derived from Hokuriku100 by seven times of continuous backcrosses, namely Koshihikari*7//(Koshihikari/Hokuriku 100 F_2_) [16]. Genetic analyses of the large grain gene and *d60* were conducted in each backcross generation. The resulting genetically segregating populations were transplanted into Shizuoka University Ohya Field, and phenotypic traits (heading date, stem length, plant type, grain length, and grain width) of all plants were investigated. For grain characteristics, we evaluated the grain diameter at the early generation of F_2_, and grain area (grain length/2 × grain width/2 × π) in the near isogenic backgrounds through backcrossing.

### 4.2. Mapping of Large Grain Gene by DNA Markers

In order to map the large grain gene, 1328 F_2_ plants of Nipponbare × Inochinoichi were used. We sampled leaves from 371 small grain homozygous F_2_ plants. The leaves were powdered while being frozen by liquid nitrogen using a Precellys 24 high-throughput bead-mill homogenizer (Bertin Technologies, Montigny-le-Bretonneux, France), and then genomic DNA was extracted using the cetyl trimethylammonium bromide (CTAB) method. A linkage analysis of large grain genes was conducted across the 12 rice chromosomes using SSR markers and SNP markers, which are polymorphic between Nipponbare and Inochinoichi. For the PCR reactions used to detect SSR markers, the mixtures were first heated to 95 °C for two minutes to denature the DNA, followed by 35 cycles of denaturing at 95 °C for 30 s, annealing at 50 °C or 55 °C for 30 s, and extension at 72 °C for 30 s. The SSR polymorphisms in the PCR products were analyzed by electrophoresis using a cartridge QIAxcel DNA Screening Kit (2400) in a QIAxcel electrophoresis apparatus (Qiagen, Hilden, Germany) at 5 kV for 10 min. SNP allele-specific TaqMan probes were designed and labeled using the fluorescent dyes FAM or HEX. The real time PCR reaction was used to amplify allele-specific fluorescence, by first heating the material to 95 °C for 30 s to denature the DNA, followed by 40 cycles of denaturing at 95 °C for 15 s, and annealing at 48 °C to 53.5 °C for 30 s.

### 4.3. Next-generation Sequencing (NGS) Analysis

Whole-genome sequencing of both Koshihikari and a large grain isogenic Koshihikari line (BC_4_F_2_), which was integrated with large grain gene derived from Inochinoichi by four times of back crosses into the genetic background of Koshihikari, were conducted. The leaves were powdered using a mortar and pestle while being frozen by liquid nitrogen. The DNA was then extracted using the CTAB method. Genomic DNA was fragmented and simultaneously tagged with the Nextera^®^ transposome (Illumina, Rockville, MD, USA) such that the peak size of fragments was approximately 500 bp. Adapter sequences, including the sequencing primers, were synthesized in both ends via PCR. After the size selection of DNA fragments using magnetic beads, the DNA library was prepared following qualitative check by Bioanalyzer 2100 system (Agilent Technologies, Inc., Palo Alto, USA), and quantitative measurement by Qubit^®^ Fluorometer (Life Technologies; Thermo Fisher Scientific, Inc., Waltham, MA, USA). The sequencing data were gained with paired-end reads using a HiSeq next-gen sequencer. The read sequences obtained were mapped using Burrows-Wheeler Aligner (BWA) software to the Nipponbare genome as a reference.

## Figures and Tables

**Table 1:** Phenotypic expression of large-grain gene integrated isogenic Koshishikari.

		*GW2* Koshihikari	*GW2* + *d60* Koshihikari	Nigata Koshihikari
Stem length (cm)		92	75	99
Weight of unpolished rice/1000 grains (g)		29.6 (×1.34)	28.8 (×1.31)	22.0
Polished rice	Taste value	80.0	80.0	81.0
	Protein	6.4	6.1	6.0
	Moisture	14.4	14.5	14.5
	Amylose	18.8	18.6	18.5
	Normal grain size	94.7	95.6	97.3
	Powdery grain	2.9	2.1	1.8
	Damaged grain	0.0	0.0	0.0
	Colored grain	0.0	0.0	0.0
	Split grain	0.2	0.3	0.3
	Crashed grain	2.2	2.0	0.6
	White degree	42.6	42.8	44.8

The large grain isogenic Koshihikari (BC_5_F_2_) showed the thousand kernel weight increased by 34%. Its taste score (80.0) was also equivalent to that of Niigata Koshihikari (81.0).

## Supplementary Materials

Supplementary materials can be found at https://www.mdpi.com/1422-0067/20/21/5442/s1. Figure S1. Complementary gamete lethal between semidwarfing gene *d60* and gamete lethal gene *gal*.
