# Supplementary figures and images for: Identification of Rice Large Grain Gene GW2 by Whole-Genome Sequencing of a Large Grain-Isogenic Line Integrated with Japonica Native Gene and Its Linkage Relationship with the Co-integrated Semidwarf Gene d60 on Chromosome 2
Source: Int J Mol Sci. 2019 Oct 31;20(21):5442. doi: 10.3390/ijms20215442 (PMC6862489; doi:10.3390/ijms20215442)

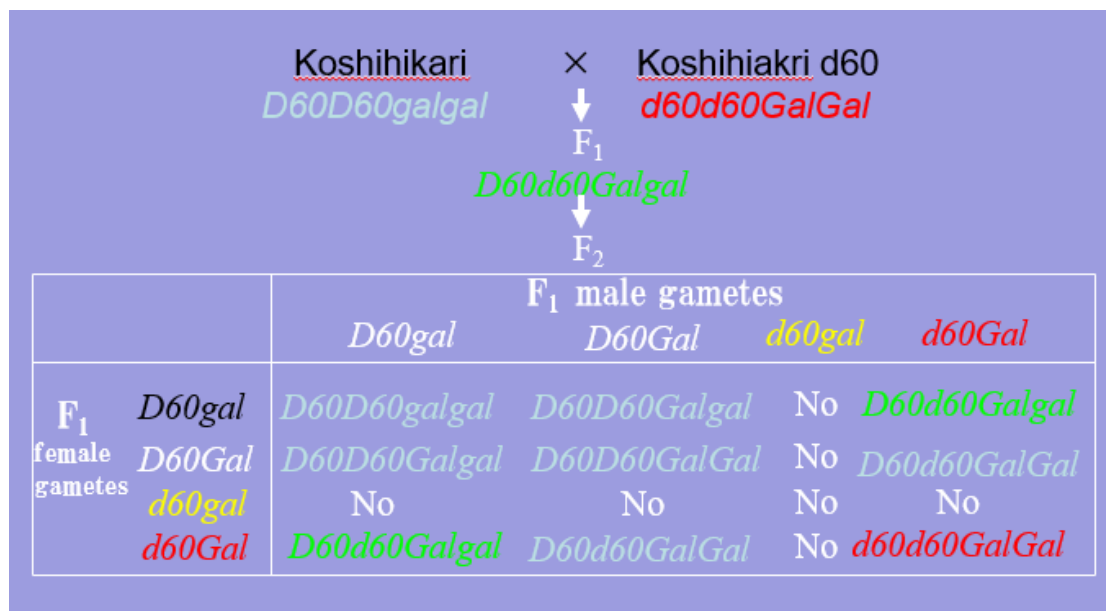

**Figure S1.** Complementary gamete lethal between semidwarfing gene *d60* and gamete lethal gene *gal*.

Supplement: Supplementary file 1 [file ijms-20-05442-s001.pdf]
